# Association Between *Helicobacter Pylori* Infection and Non-alcoholic Fatty Liver Disease, Hepatic Adipose Deposition and Stiffness in Southwest China

**DOI:** 10.3389/fmed.2021.764472

**Published:** 2021-12-24

**Authors:** Ying Liu, Dongyu Li, Yuping Liu, Ping Shuai

**Affiliations:** ^1^Health Management Center, Sichuan Provincial People's Hospital, University of Electronic Science and Technology of China, Chengdu, China; ^2^Chinese Academy of Sciences Sichuan Translational Medicine Research Hospital, Chengdu, China

**Keywords:** *Helicobacter pylori*, non-alcoholic fatty liver disease, transient elastography, liver stiffness measurement, fat attenuation parameter

## Abstract

**Background:** Both nonalcoholic fatty liver disease (NAFLD) and *Helicobacter pylori* (*H. pylori*) infection have high prevalence worldwide, and the relationship between both remains controversial. We try to investigate whether *H. pylori* infection is associated with NAFLD and increased liver fat deposition and stiffness in this cross-sectional study.

**Methods:** The physical examination data of 5,665 subjects were obtained from February 2018 to June 2019 in this study. Clinical and biochemical data were collected. NAFLD was diagnosed using abdominal color Doppler ultrasonography. Liver steatosis and stiffness were understood by two parameters of transient elastography (TE): fat attenuation parameter (FAP) and liver stiffness measurement (LSM). *H. pylori* infection was determined using the ^13^C urea breath tests.

**Results:** The total prevalence of NAFLD and *H. pylori* infection was 30.2 and 37.0%, respectively. In men, the prevalence of NAFLD and the levels of FAP and LSM in *H. pylori*-positive group were significantly higher than *H. pylori*-negative group (all *p* < 0.01), but no significant difference was found in women. In men, the infection rate of *H. pylori* in NAFLD group and LSM ≥ 7.4 kPa group was significantly higher than control group. Multivariate logistic regression analysis revealed that *H. pylori* infection was not independently associated with NAFLD and FAP ≥ 240 dB/m. However, *H. pylori* infection was associated with LSM ≥ 7.4 kPa in men.

**Conclusions:** Our study suggests that *H. pylori* infection is not significantly associated with NAFLD and elevated liver steatosis, whereas it may be the risk factor of elevated liver stiffness in men.

## Introduction

Non-alcoholic fatty liver disease (NAFLD) is a worldwide liver disease in the contemporary society, and the global prevalence of NAFLD is estimated around 25% (1); the situation is similar in China (2). Considering the high incidence rate of NAFLD and its potential risk of adverse effects such as liver fibrosis and cirrhosis, liver failure, and even hepatocellular carcinoma (HCC), it is necessary to explore the pathogenesis of NAFLD and intervene actively.

Although the etiology of NAFLD has not been fully elucidated, it is closely related to a variety of metabolic disorders caused by insulin resistance (IR) and is even considered to be the liver manifestation of metabolic syndrome (MetS) (3). Recently, the relationship between NAFLD and microbes has attracted much attention. *Helicobacter pylori* (*H. pylori*), which is estimated to infect approximately 50% of the world population, has been paid attention to its extragastric manifestations. It is also to be found that *H. pylori* infection plays a potential role in IR development, which makes researchers who are interested in exploring the correlation between *H. pylori* infection and NAFLD (4).

Some studies tried to discuss the relationship between *H. pylori* infection and NAFLD. In a meta-analysis, a significantly increased risk of NAFLD among the patients with *H. pylori* infection had been found (5). However, controversial results also remained (6). First, the prevalence of NAFLD and *H. pylori* infection may not be the same in different regions, races, social customs, and eating habits, which makes the results of their correlation inconsistent. The comprehensive analysis of data from multiple regions worldwide is needed. Second, NAFLD is usually diagnosed according to the color Doppler ultrasonography in previous studies, and there may be some artificial bias. Furthermore, for liver fibrosis, which is of great significance in the pathological process of NAFLD, whether *H. pylori* infection is related to its progression is still lacking relevant research. Considering the difficulty in obtaining a number of liver pathological specimens from the general population infected with *H. pylori*, it is feasible to obtain the relevant epidemiological data through a noninvasive examination that can quantitatively understand the steatosis and fibrosis of liver.

Transient elastography (TE) is such a detection technology that measures liver stiffness using mechanical and/or ultrasound shear wave propagation through hepatic parenchyma with high accuracy. According to liver stiffness measurement (LSM) and fat attenuation parameter (FAP) from TE, we can get the quantitative assessment of liver fibrosis and steatosis, which had a good consistency with liver biopsy (7, 8). Several guidelines recommend the use of TE to assess liver fat deposition and fibrosis (9–11).

Thus, we conducted this study not only according to abdominal color Doppler ultrasonography but also through TE, to investigate the prevalence of *H. pylori* infection in different NAFLD status and steatosis, stiffness levels of liver. To our knowledge, we have not found any similar study before. Quantitative analysis of NAFLD in this study may provide more helpful results to accurately understand the relationship between NAFLD and *H. pylori* infection.

## Materials and Methods

### Subjects

The data were obtained from the Health Management Center, Sichuan Provincial People's Hospital. All the subjects were asked to complete a medical history questionnaire, followed by physical examination (height, body weight, blood pressure, and circumference of waist, hip, and neck), and laboratory examination (routine blood test, liver and kidney function, fasting plasma glucose (FPG), hemoglobin A1c (HbA1c), uric acid, total cholesterol, triglycerides, low-density lipoprotein cholesterol (LDL-C), high-density lipoprotein cholesterol (HDL-C), and hepatitis B virus markers), abdominal ultrasonography, chest imaging (X-ray or CT), TE FibroTouch, and ^13^C urea breath test.

The metabolic syndrome (MetS) was defined by the presence of at least three of the following five metabolic abnormalities, based on the Guidelines for the Prevention and Treatment of Type 2 Diabetes in China (2017 edition): waist circumference ≥ 90 cm for men and ≥ 85 cm for women; FPG ≥ 6.1 mmol/L and/or type 2 diabetes mellitus (T2DM) was previously diagnosed and treated; blood pressure ≥ 130/85 mmHg and/or hypertension was previously diagnosed and treated; fasting triglyceride ≥ 1.7 mmol/L; fasting HDL cholesterol <1.04 mmol/L.

Subjects were excluded if they had (a) alcoholic abuse, had a daily consumption ≥ 30 g for men and ≥ 20 g for women, (b) evidence of suffered from hepatitis B virus, hepatitis C virus, or human immunodeficiency virus (HIV), (c) a history of using drugs that can cause hepatic steatosis, such as corticosteroids, amiodarone, estrogen, and so on, (d) severe malnutrition, parenteral nutrition, and other end-stage diseases or cancers, (e) contraindications for FibroTouch examination (i.e., ascites, pregnancy, heart pacemakers, and other implanted materials).

The study protocol was approved by the ethics committees of the Sichuan Provincial People's Hospital, according to Declaration of Helsinki (2013) and its later amendments or comparable ethical standards [approval number 177(2017)].

### Assessment of NAFLD

Nonalcoholic fatty liver disease was diagnosed using abdominal ultrasonography (Philips IU elite, Siemens, Germany), the probe frequency was 1–5 MHz, and the center frequency was 3.5 MHz, performed by a doctor at or above the rank of the attending physician from the imaging center of our hospital. According to the diagnostic criteria issued by Chinese Medical Association (12), hepatic steatosis was defined based on the presence of at least two of the three following findings: the near-field echo of liver was diffuse enhanced and stronger than that of kidney; poor visualization of intrahepatic duct structure; far-field echo of liver attenuated gradually.

### Transient Elastography

The new generation of TE series FibroTouch (Wuxi Hisky Medical Technology, Beijing, China) was applied. Two trained physicians used FibroTouch according to the operations manual, blinded to the patients' clinical data. LSM analyses were expressed in kilopascals (kPa) and FAP was expressed in decibels per meter (dB/m). Only if 10 successful measurements were obtained, an IQR/M ratio of LSM and FAP <30% and a success rate of > 60% were considered reliable and then used for analysis. A cutoff value of FAP ≥ 240 dB/m and LSM ≥ 7.4 kPa was considered for elevated liver steatosis and stiffness, respectively.

### *H. pylori* Infection Test

*Helicobacter pylori* infection was determined using the ^13^C urea breath tests (Beijing Boran Pharmaceutical Co., Ltd. Beijing, China). We followed a standardized procedure for the sample collection. All subjects fasted overnight for more than 8 h, maintained normal breath, inserted the straw into the bottom of one sample tube, and exhaled slowly into the sample tube through the straw for 4 to 5 s. Thereafter, they pulled the straw out, tightened the cap immediately, and this was considered as the 0-min sample. Then, the subjects took another bottle with urea ^13^C granules and 80 to 100 mL cold drinking water, rested for 30 min, and then collected the breath sample again. The two collected gas samples were tested for ^13^CO_2_, and δ‰ was used to represent the determination result; δ‰ = (isotopic abundance of ^13^C for the test sample – isotopic abundance of ^13^C for reference sample) × 1000/the isotopic abundance ^13^C for reference sample. The detection value was defined as δ‰ measured at 30 min subtracted from that measured at 0 min. *H. pylori* infection was considered positive when the detection value was ≥ 4.0.

### Statistical Analysis

Statistical analysis was performed using IBM SPSS 21.0 (IBM Corp., NY, USA). Continuous data are expressed as mean ± standard deviation (SD) for normally distributed data and median with 25th and 75th percentile for non-normally distributed data. Categorical data are expressed in percentages. The significance of differences was tested using either Student's *t*-test (for continuous variables) or chi-squared test (for categorical variables). Univariable and multivariable regression models were performed using the logistic regression analysis, and the results were expressed as odds ratios (*ORs*) and 95% confidence intervals (*CIs*). A *p*-value < 0.05 was considered statistically significant.

## Results

### Baseline of the Study Population Characteristics

The total clinical and laboratory baseline characteristics of the 5,665 subjects (3,089 men and 2,576 women) in the study are shown in Table 1, with a mean age of 49.07 ± 10.17 years. 36.4% had hypertension or SBP ≥ 130 mmHg and/or DBP ≥ 85 mmHg, 11.0% had T2DM or FPG ≥ 6.1 mmol/L, 32.7% had dyslipidemia, 35.5% had abdominal overweight, and 18.1% met the diagnostic criteria of MetS. The prevalence of the items mentioned above was higher in men than that in women, and the differences were statistically significant. The total infection rate of *H*. pylori was 37.0%, which was higher in men than that in women, (38.1 vs. 35.7%), but no significant difference was found (*p* = 0.057).

**Table 1:** Baseline characteristics of the study population (*n* = 5,665).

**Variables**	**All**	**Male**	**Female**	***p*** **-value**
	**(***n*** = 5,665)**	**(***n*** = 3,089)**	**(***n*** = 2,576)**	
**Demographic data**				
Sex (female), *n* (%)	2576 (45.5)			
Age (years)	49.07 ± 10.17	49.06 ± 10.41	49.10 ± 9.89	0.882
Hypertension†, *n* (%)	2060 (36.4)	1311 (42.4)	749 (29.1)	<0.001
Hyperglycemia†, *n* (%)	824 (11.0)	414 (13.4)	167 (6.5)	<0.001
Dyslipidemia†, *n* (%)	1852 (32.7)	1596 (51.7)	541 (21.0)	<0.001
Waist circumferences ≥ 90 cm in men and ≥ 85 cm in women, *n* (%)	2663 (35.5)	1251 (40.5)	601 (23.3)	<0.001
MetS^†^, *n* (%)	1026 (18.1)	815 (26.4)	211 (8.2)	<0.001
Smoking, *n* (%)	1291 (22.8)	1243 (40.2)	48 (1.9)	<0.001
Anthropometric data				
Body weight (kg)	64.58 ± 11.87	70.84 ± 10.81	57.06 ± 8.14	<0.001
Height (cm)	162.58 ± 8.24	167.71 ± 6.24	156.42 ± 5.75	<0.001
BMI (kg/m2)	24.32 ± 3.31	25.14 ± 3.20	23.33 ± 3.16	<0.001
Waist circumferences (cm)	83.57 ± 9.93	87.77 ± 8.93	78.54 ± 8.66	<0.001
Hip circumferences (cm)	94.88 ± 6.24	96.45 ± 6.07	92.99 ± 5.92	<0.001
Waist-Hip ratio	0.88 ± 0.07	0.91 ± 0.06	0.84 ± 0.07	<0.001
Neck circumferences (cm)	35.20 ± 3.65	37.61 ± 2.70	32.30 ± 2.27	<0.001
Laboratory data				
ALT (U/L)	23 (16, 34)	29 (21, 41)	18 (14, 25)	<0.001
AST (U/L)	28.49 ± 9.79	29.88 ± 10.65	26.82 ± 8.34	<0.001
GGT (U/L)	24 (16, 40)	31 (21, 51)	17 (13,25)	<0.001
Fasting glucose (mmol/L)	5.21 ± 1.52	5.32 ± 1.73	5.07 ± 1.19	<0.001
HbA1c (%)	5.56 ± 0.88	5.64 ± 0.96	5.45 ± 0.75	<0.001
Total cholesterol (mmol/L)	4.96 ± 0.93	4.97 ± 0.93	4.95 ± 0.92	0.258
Triglycerides (mmol/L)	1.35 (0.95, 2.02)	1.60 (1.14, 2.37)	1.11 (0.82, 1.57)	<0.001
LDL cholesterol (mmol/L)	2.87 ± 0.76	2.93 ± 0.78	2.80 ± 0.74	<0.001
HDL cholesterol (mmol/L)	1.37 ± 0.33	1.23 ± 0.27	1.54 ± 0.32	<0.001
Uric acid (μmol/L)	336.54 ± 87.95	385.18 ± 78.65	278.22 ± 57.94	<0.001
Platelet count (10^9^/L)	196.84 ± 61.06	193.16 ± 59.53	201.26 ± 62.57	<0.001
*H. pylori*-positive, *n* (%)	2097 (37.0)	1178 (38.1)	919 (35.7)	0.056
NAFLD, *n* (%)	1710 (30.2)	1279 (41.4)	431 (16.7)	<0.001

*T2DM, type 2 diabetes mellitus; BMI, body mass index; ALT, alanine aminotransferase; AST, aspartate aminotransferase; GGT, gamma-glutamyltransferase; HbA1c, hemoglobin A1c*.

† *According to the Guidelines for the Prevention and Treatment of Type 2 Diabetes in China (2017 edition): (1) hypertension: hypertension previously diagnosed or SBP ≥ 130 mmHg and/or DBP ≥ 85 mmHg; (2) T2DM: T2DM previously diagnosed and/or FPG level ≥ 6.1 mmol/L; (3) dyslipidemia: fast triglyceride levels ≥ 1.7 mmol/L and/or fast HDL cholesterol <1.04 mmol/L*.

### Prevalence of NAFLD and Levels of FAP and LSM in Different *H. pylori* Status

Subjects were categorized based on their sex to investigate the differences in the prevalence of NAFLD and levels of FAP and LSM in different *H. pylori* status. In men, the prevalence of NAFLD in *H. pylori*-positive group was significantly higher than that in *H. pylori*-negative group (44.5 vs. 39.5%, *p* <0.01). The levels of FAP and LSM in *H. pylori*-positive groups were also significantly higher than that in *H. pylori*-negative groups (all *p* < 0.01). However, in women, no significant differences were found in the above three indicators between *H. pylori*-positive and *H. pylori*-negative groups, as shown in Table 2.

**Table 2:** Comparison of NAFLD prevalence and FAP, LSM levels in different H. pylori status.

**Variables**	**Male**				**Female**			
	***H. pylori*** **(-)**	***H. pylori*** **(+)**	**χ^2^ or *t* value**	***p*** **-value**	***H. pylori*** **(-)**	***H. pylori*** **(+)**	**χ^2^ or ***t*** value**	***P*** **value**
NAFLD [*n*(%)]								
yes	1156(60.5)	654(55.5)	7.432	0.006	1387(83.7)	758(82.5)	0.636	0.425
no	755(39.5)	524(44.5)			270(16.3)	161(17.5)		
FAP (dB/m)	248.38 ± 32.97	252.01 ± 34.47	−2.889	0.004	229.00 ± 31.42	230.92 ± 31.93	−1.481	0.139
LSM (kPa)	6.20 ± 1.69	6.39 ± 1.78	−2.969	0.003	5.67 ± 1.49	5.76 ± 1.51	−1.442	0.149

*NAFLD, nonalcoholic fatty liver disease; FAP, fat attenuation parameter; LSM, liver stiffness measurement*.

### The Prevalence of *H. pylori* Infection in Different NAFLD, FAP, and LSM Status

**Figure 1:**
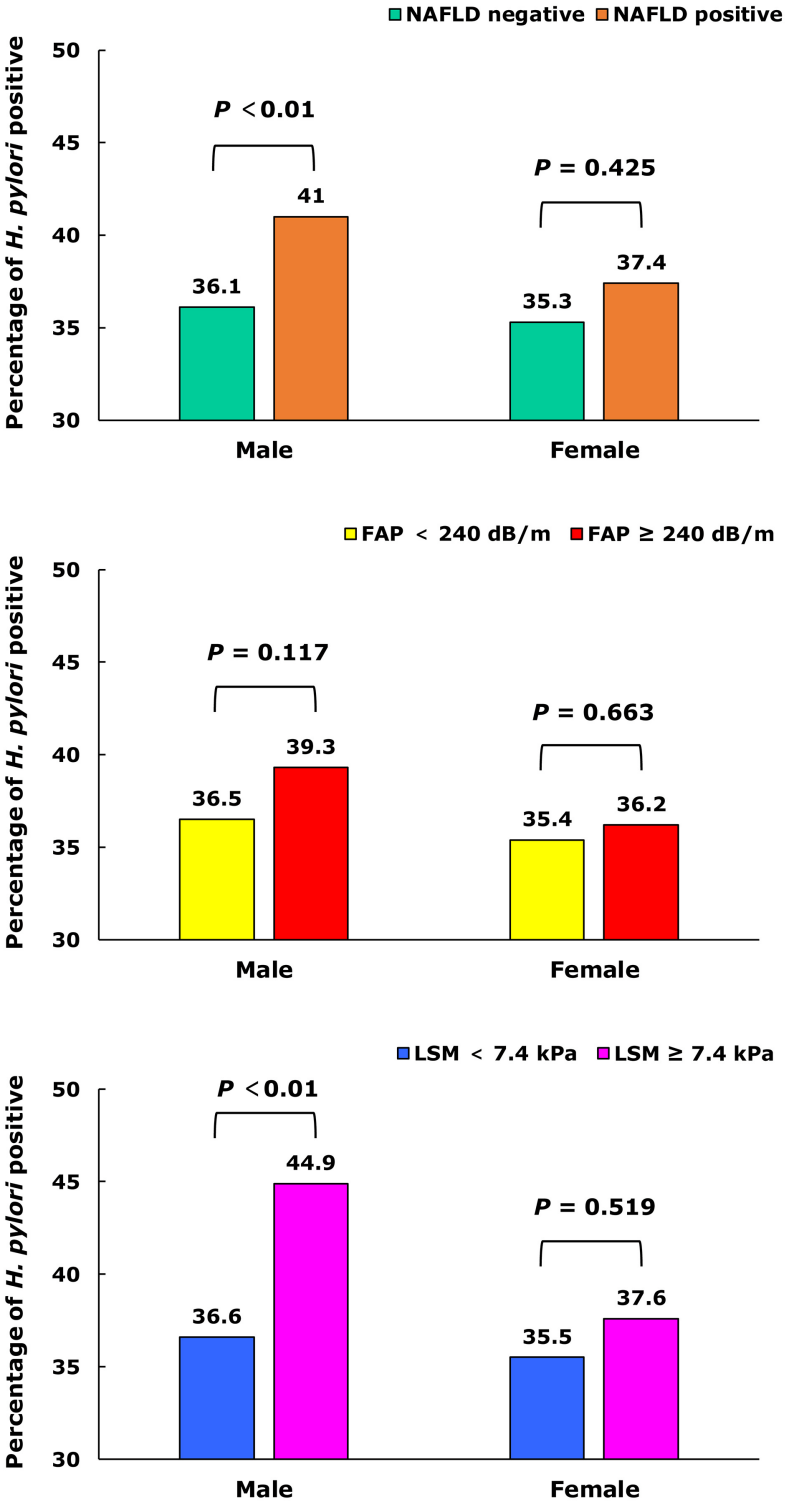
The prevalence of *H. pylori* in different NAFLD, FAP, and LSM status.

In men, the prevalence of *H. pylori* infection was significantly higher in NAFLD and LSM ≥ 7.4 kPa groups than the corresponding control groups (41.0 vs. 36.1%, 44.9% vs. 36.6%, respectively, all *p* < 0.01), but it was not found in women. There was no significant difference in the prevalence of *H. pylori* infection between FAP ≥ 240 dB/m group and control group in both men and women (all *p* > 0.05), as shown in Figure 1.

### Multivariate Logistic Regression Analysis of NAFLD and Increased Levels of FAP and LSM

As shown in Tables 3–5, stratified analyses were performed for age, sex, and dyslipidemia, to explore the association between *H. pylori* infection with NAFLD and increased levels of FAP and LSM. After adjusting for confounding factors in different models, *H. pylori* infection was not found to be a risk factor for NAFLD and FAP ≥ 240 dB/m. However, *H. pylori* infection was revealed to be the risk factor for LSM ≥ 7.4 kPa in men and above 50-year-old subjects.

**Table 3:** Multivariate logistic regression analysis of the association between *H. pylori* infection and NAFLD, stratified by age, sex, and dyslipidemia.

	**Crude model**	**Model 1**	**Model 2**	**Model 3**
Age (years)				
< 50	1.152 (0.977–1.359)	1.068 (0.881–1.293)	0.959 (0.770–1.195)	0.973 (0.776–1.220)
≥ 50	1.230 (1.043–1.452)	1.136 (0.949–1.359)	1.017 (0.835–1.240)	1.029 (0.841–1.260)
Sex				
Male	1.227 (1.059–1.421)	1.168 (0.996–1.369)	1.023 (0.857–1.223)	1.048 (0.873–1.257)
Female	1.091 (0.881–1.352)	0.992 (0.787–1.250)	0.924 (0.711–1.199)	0.901 (0.690–1.177)
Dyslipidemia^§^				
No	1.227 (1.020–1.476)	1.172 (0.966–1.422)	1.017 (0.818–1.264)	1.014 (0.813–1.266)
Yes	1.127 (0.946–1.342)	1.056 (0.879–1.270)	0.957 (0.786–1.167)	0.990 (0.808–1.214)

*Model 1: adjusted for sex, age, Met; Model 2: adjusted for sex, age, BMI, hypertension†, hyperglycemia^‡^, dyslipidemia^§^; Model 3: adjusted for sex, age, BMI, hypertension†, hyperglycemia^‡^, dyslipidemia^§^, UA, ALT, AST, GGT, smoking. †hypertension: blood pressure ≥ 130/85 mmHg and/or hypertension previously diagnosed and treated. ^‡^hyperglycemia: FPG level ≥ 6.1 mmol/L and/or T2DM previously diagnosed and treated*.

§ *dyslipidemia: fasting triglyceride levels ≥ 1.7 mmol/L; fasting HDL-cholesterol <1.04 mmol/L*.

**Table 4:** Multivariate logistic regression analysis of the association between *H. pylori* infection and FAP ≥ 240 dB/m, stratified by age, sex, and dyslipidemia.

	**Crude model**	**Model 1**	**Model 2**	**Model 3**
Age (years)				
< 50	1.089 (0.938–1.265)	1.019 (0.862–1.204)	0.907 (0.744–1.106)	0.902 (0.739–1.100)
≥ 50	1.107 (0.946–1.294)	1.026 (0.870–1.211)	0.876 (0.723–1.061)	0.870 (0.718–1.055)
Sex				
Male	1.126 (0.971–1.305)	1.067 (0.912–1.248)	0.907 (0.755–1.089)	0.912 (0.758–1.096)
Female	1.038 (0.877–1.229)	0.963 (0.805–1.151)	0.857 (0.695–1.056)	0.842 (0.682–1.040)
Dyslipidemia^§^				
No	1.031 (0.893–1.191)	0.990 (0.854–1.148)	0.833 (0.700–0.992)	0.827 (0.694–0.985)
Yes	1.155 (0.955–1.397)	1.077 (0.881–1.315)	1.978 (0.780–1.226)	0.985 (0.784–1.237)

*Model 1: adjusted for sex, age, Met; Model 2: adjusted for sex, age, BMI, hypertension†, hyperglycemia^‡^, dyslipidemia^§^; Model 3: adjusted for sex, age, BMI, hypertension†, hyperglycemia^‡^, dyslipidemia^§^, UA, ALT, AST, GGT, smoking. †hypertension: blood pressure ≥ 130/85 mmHg and/or hypertension previously diagnosed and treated. ^‡^hyperglycemia: FPG level ≥ 6.1 mmol/L and/or T2DM previously diagnosed and treated*.

§ *dyslipidemia: fasting triglyceride levels ≥ 1.7 mmol/L; fasting HDL-cholesterol <1.04 mmol/L*.

**Table 5:** Multivariate logistic regression analysis of the association between *H. pylori* infection and LSM ≥ 7.4 kPa, stratified by age, sex, and dyslipidemia.

	**Crude model**	**Model 1**	**Model 2**	**Model 3**
Age (years)				
< 50	1.239 (0.993–1.545)	1.184 (0.944–1.485)	1.100 (0.871–1.389)	1.109 (0.877–1.401)
≥ 50	1.359 (1.106–1.670)	1.293 (1.049–1.595)	1.240 (1.002–1.535)	1.251 (1.010–1.551)
Sex				
Male	1.413 (1.177–1.697)	1.360 (1.130–1.636)	1.263 (1.045–1.526)	1.282 (1.060–1.552)
Female	1.094 (0.833–1.436)	1.033 (0.783–1.364)	1.011 (0.763–1.340)	1.016 (0.766–1.348)
Dyslipidemia^§^				
No	1.287 (1.038–1.596)	1.236 (0.993–1.539)	1.169 (0.936–1.460)	1.181 (0.945–1.477)
Yes	1.314 (1.062–1.626)	1.259 (1.015–1.563)	1.181 (0.947–1.474)	1.199 (0.960–1.499)

*Model 1: adjusted for sex, age, Met; Model 2: adjusted for sex, age, BMI, hypertension†, hyperglycemia^‡^, dyslipidemia^§^; Model 3: adjusted for sex, age, BMI, hypertension†, hyperglycemia^‡^, dyslipidemia^§^, UA, ALT, AST, GGT, smoking. †hypertension: blood pressure ≥ 130/85 mmHg and/or hypertension previously diagnosed and treated. ^‡^hyperglycemia: FPG level ≥ 6.1 mmol/L and/or T2DM previously diagnosed and treated*.

§ *dyslipidemia: fasting triglyceride levels ≥ 1.7 mmol/L; fasting HDL-cholesterol <1.04 mmol/L*.

## Discussion

As one of the main causes of chronic liver diseases, exploring the etiology of NAFLD has become a hot spot. The prevalent pathogenic model of NAFLD is that of “parallel multiple hit,” and *H. pylori* infection may be one of the “hit” (13). Cindoruk et. al found that *H. pylori* 16S rDNA was discovered from patient with NAFLD by liver biopsy (14). Since then, scholars had discussed the relationship between *H. pylori* and NAFLD and proposed some mechanisms between the both.

The pathogenetic link between NAFLD and *H. pylori* was in debate and clinical data were limited. IR was focused on to be the underlying mechanism between *H. pylori* and NAFLD. Polyzos et al. first reported that anti-*H. pylori* IgG levels were significantly higher in patients with liver biopsy-proven NAFLD, which indicate that *H. pylori* infection might contribute to NAFLD directly or indirectly *via* IR (13). The patients who suffered from *H. pylori* infection had increased expression of inflammatory factors such as IL-1, IL-6, and TNF-a, which reduced insulin sensitivity (4). Some hormones such as leptin, adiponectin, fetuin-A have also been found the changes in IR promotion in patients with *H. pylori* infection (6). Furthermore, NAFLD also presented increased intestinal permeability, and that liver might be damaged by bacteria and its toxins contained in the portal flow in patients with NAFLD. Based on this, it could be hypothesized that those hormones and pro-inflammatory cytokines induced by *H. pylori* may also gain into liver *via* portal vein and contribute to the pathogenesis of NAFLD (15). Additionally, researchers who reported the changes in homeostatic model assessment of IR, fatty liver index, and echographic liver pattern in patient with *H. pylori* had found that the metabolic profile improved after the treatment for *H. pylori* eradication (16). However, studies for an association between *H. pylori* infection and NAFLD had also produced contradictory results (17). Considering that both *H. pylori* infection and NAFLD were multifactorial pathogenesis, more data from multiple regions around the world were needed to explore the relationship between the both.

Previous studies usually used color Doppler ultrasound to diagnose NAFLD, and this qualitative diagnosis method might have artificial bias. To better understand the correlation between *H. pylori* infection and NAFLD, we used TE to evaluate the degree of liver adipose deposition while defining NAFLD by color ultrasound. In our study, the results showed that in men, the prevalence of NAFLD and FAP level in the *H. pylori*-positive groups were all higher than *H. pylori*-negative groups. In women, the *H. pylori*-positive rate in the NAFLD group was also higher than non-NAFLD group whereas there was no significant difference in the *H. pylori*-positive rate between groups of FAP ≥ 240 dB/m and <240 dB/m. According to multiple regression analysis, *H. pylori* was not a risk factor for NAFLD and FAP ≥ 240 dB/m in both men and women.

Our findings were consistent with some previous studies. Okushin et al. reported that no significant relationship between NAFLD and *H. pylori* seropositivity was found in a large Japanese cross-sectional study of 13,737 participants (18). Baeg et al. also failed to find a correlation between *H. pylori* and NAFLD by analyzing the data of 3,663 South Koreans (19). Even in studies that had positive results, the *OR* values were not significant (20–23). In our view, the results mentioned above may illustrate a problem, even if *H. pylori* infection does have an impact on the pathological development of NAFLD, its role may be limited, with relatively small weights compared with other risk factors of NAFLD.

Although we did not find positive correlation between *H. pylori* infection and NAFLD, *H. pylori* infection was found to be associated with high LSM value, suggesting that *H. pylori* infection may be a risk factor for increased liver stiffness. After further multivariate regression analysis stratified by age, sex, and dyslipidemia, the results revealed the association maintained in men, but not among women.

In 2009, Goo et al. reported that in the animal model of carbon tetrachloride (CCl_4_)-induced hepatic cirrhosis, significant increase in the fibrotic score and also in serum ALT and AST levels was shown in the CCl_4_+*H. pylori* group compared with that in the CCl_4_-treated group. In addition, immunohistochemical study against *H. pylori* shows positive antigen fragments in the liver of the infected group, suggested that *H. pylori* infection could be an important infectious factor contributing to the development of liver cirrhosis (24). Furthermore, liver fibrogenesis was due to an imbalance between fibrogenesis and fibrolysis, and hepatic stellate cells (HSCs) were the pivot in this process. The results of animal experiments by Ki et al. suggested that *H. pylori* endotoxins that transported in the portal vein to the liver, coupled with liver injury by CCl_4_, might accelerate hepatic fibrosis through increasing TGF-b1-inducing proinflammatory signaling mediated by ERK and NF-kB in HSCs (25). It had also been reported in humans supporting that *H. pylori* was associated with the progression of liver fibrosis. Polyzos et al. reported that thirteen adult patients with biopsy-proven non-alcoholic steatohepatitis underwent a ^13^C urea breath test, and the patients with *H. pylori*-positive received eradication therapy. The result shows that *H. pylori* eradication had no long-term effect on hepatic steatosis, but shows a trend toward improvement in NAFLD fibrosis score (26).

However, the studies mentioned above were either animal models or the sample size which was too small, and the animal models established by chemical drugs were also different from the natural course of *H. pylori* infection in general human population. Considering the difficulty of obtaining a large number of liver samples from the general population infected with *H. pylori*, it is feasible to obtain relevant epidemiological data first. Currently, there is a lack of large sample studies on the correlation between *H. pylori* with the progression of early liver fibrosis in the general population. Our results supported the positive correlation between *H. pylori* infection and liver fibrosis at the epidemiological level and suggested that *H. pylori* may have different pathogenic risks in liver fat deposition and liver fibrosis progression in men. It was particularly important to understand this, because for a series of pathological processes of NAFLD, fat deposition could be reversed. However, liver fibrosis was somewhat difficult to reverse, and its impact on the development of liver disease was more profound.

The limitation of this study was the lack of comparison of liver pathological findings. The definition of NAFLD was determined by ultrasonography but not by liver biopsy. However, to some extent, this was compensated by TE, which had a good consistency with liver biopsy. Second, our study was cross-sectional, lack of intervention comparison, and the results only reflected epidemiological links, but failed to draw pathological and clinical conclusions. Even though we have found that *H. pylori* infection may be associated with increased liver stiffness in men, considering the gender difference of this correlation, we could not distinguish whether this was directly related to the interaction between them, or only reflected the differences of social behavior and lifestyle between men and women. A well-designed prospective study is warranted to clarify whether there is a causative link between them in the future.

In conclusion, our study suggests that *H. pylori* infection is not significantly associated with NAFLD and elevated liver steatosis, whereas it may be the risk factor of elevated liver stiffness in men. If this correlation is confirmed at the pathophysiological level of clarify the causative link between them in the future, eradication of *H. pylori* may be considered in therapeutic strategies of the relevant male population.

## Data Availability Statement

The raw data supporting the conclusions of this article will be made available by the authors, without undue reservation.

## Ethics Statement

The studies involving human participants were reviewed and approved by the Ethics Committees of the Sichuan Provincial People's Hospital. Written informed consent for participation was not required for this study in accordance with the national legislation and the institutional requirements.

## Author Contributions

YL and PS designed the study. YL and DL performed the data collection and analysis. YL and PS wrote the manuscript. YpL assisted in data collection and document writing. All authors read and approved the manuscript.

## Funding

This work was supported by Key Research and Development Projects of the Ministry of Science and Technology, China, No. 2017YFC0113901.

## Conflict of Interest

The authors declare that the research was conducted in the absence of any commercial or financial relationships that could be construed as a potential conflict of interest.

## Publisher's Note

All claims expressed in this article are solely those of the authors and do not necessarily represent those of their affiliated organizations, or those of the publisher, the editors and the reviewers. Any product that may be evaluated in this article, or claim that may be made by its manufacturer, is not guaranteed or endorsed by the publisher.
